# The Unexpected Effects of Beneficial and Adverse Social Experiences during Adolescence on Anxiety and Aggression and Their Modulation by Genotype

**DOI:** 10.3389/fnbeh.2016.00097

**Published:** 2016-05-26

**Authors:** Neele Meyer, S. Helene Richter, Rebecca S. Schreiber, Vanessa Kloke, Sylvia Kaiser, Klaus-Peter Lesch, Norbert Sachser

**Affiliations:** ^1^Department of Behavioural Biology, University of MuensterMuenster, Germany; ^2^Muenster Graduate School of Evolution, University of MuensterMuenster, Germany; ^3^Laboratory of Translational Neuroscience, Division of Molecular Psychiatry, Department of Psychiatry, Psychosomatics, and Psychotherapy, University of WuerzburgWuerzburg, Germany

**Keywords:** adolescence, anxiety-like behavior, aggressiveness, serotonin transporter, coping with challenge, social experience, adversity, 5-HTT knockout mice

## Abstract

Anxiety and aggression are part of the behavioral repertoire of humans and animals. However, in their exaggerated form both can become maladaptive and result in psychiatric disorders. On the one hand, genetic predisposition has been shown to play a crucial modulatory role in anxiety and aggression. On the other hand, social experiences have been implicated in the modulation of these traits. However, so far, mainly experiences in early life phases have been considered crucial for shaping anxiety-like and aggressive behavior, while the phase of adolescence has largely been neglected. Therefore, the aim of the present study was to elucidate how levels of anxiety-like and aggressive behavior are shaped by social experiences during adolescence and serotonin transporter (5-HTT) genotype. For this purpose, male mice of a 5-HTT knockout mouse model including all three genotypes (wildtype, heterozygous and homozygous 5-HTT knockout mice) were either exposed to an adverse social situation or a beneficial social environment during adolescence. This was accomplished in a custom-made cage system where mice experiencing the adverse environment were repeatedly introduced to the territory of a dominant opponent but had the possibility to escape to a refuge cage. Mice encountering beneficial social conditions had free access to a female mating partner. Afterwards, anxiety-like and aggressive behavior was assessed in a battery of tests. Surprisingly, unfavorable conditions during adolescence led to a decrease in anxiety-like behavior and an increase in exploratory locomotion. Additionally, aggressive behavior was augmented in animals that experienced social adversity. Concerning genotype, homozygous 5-HTT knockout mice were more anxious and less aggressive than heterozygous 5-HTT knockout and wildtype mice. In summary, adolescence is clearly an important phase in which anxiety-like and aggressive behavior can be shaped. Furthermore, it seems that having to cope with challenge during adolescence instead of experiencing throughout beneficial social conditions leads to reduced levels of anxiety-like behavior.

## Introduction

Anxiety and aggressiveness are essential traits to optimally cope with environmental challenges and trigger adaptive responses. However, in their exaggerated form, i.e., if anxiety-like and aggressive behavior occur out of context and control, they are often associated with psychopathologies like generalized anxiety disorders, phobias, depression and antisocial personality disorder (Gross and Hen, 2004; Haller and Kruk, 2006).

Both anxiety and aggressiveness can be shaped by environmental and genetic factors (Kendler, 1995; Hovatta and Barlow, 2008; Anholt and Mackay, 2012; Provençal et al., 2015). Concerning the environment, adverse experiences especially during early phases in life have been studied extensively. Regarding anxiety, mainly anxiogenic effects have been found; i.e., childhood maltreatment leads to an increase in anxiety disorders and heightened depression risk in humans (Kaufman et al., 2004; Hovens et al., 2010) while in rodents low levels of maternal care are frequently associated with higher levels of anxiety-like behavior (Caldji et al., 1998; Francis, 1999; Calatayud et al., 2004; Carola et al., 2008; Wei et al., 2010; Ishikawa et al., 2015). Considering the effect of environmental factors during early phases in life on aggressiveness, childhood adversity frequently leads to higher expression of aggressive behavior in later life (Carver et al., 2014; Haller et al., 2014; Provençal et al., 2015). Similar results are found in rodents; i.e., rats that experienced maternal separation show increased levels of aggressiveness in adulthood (Veenema et al., 2006). Over the last few years, adolescence emerged as another possible sensitive period in which the behavior of an individual can be shaped (Spear, 2000; Sachser et al., 2011, 2013). Adolescence is marked as the gradual transition from childhood to adulthood and comprises the time around puberty (sexual maturation), but is not limited to it (Spear, 2000). There are some indications that also anxiety and aggressiveness can be modulated in this period. For example, adverse life events during adolescence lead to higher levels of anxiety in humans (Tsoory et al., 2007) and mice (Chaby et al., 2015). Moreover, the social experiences during this life phase, for instance social instability or social subjugation, alter aggressive behavior in a number of rodent species (Wommack et al., 2003; Cumming et al., 2014; Hennessy et al., 2015).

Concerning genetic factors, especially genes influencing serotonergic (5-HT) neurotransmission have been shown to affect anxiety and aggressiveness (Lesch and Merschdorf, 2000; Caramaschi et al., 2007; Audero et al., 2013; Olivier, 2015). In particular, the serotonin transporter (5-HTT) is a crucial regulator of the duration of the serotonergic response by removing serotonin from the synaptic cleft. In human and non-human primates 5-HTT expression and function is influenced by a variation on the 5-HTT-linked polymorphic region (5-HTTLPR) and is reported to influence aggression as well as anxiety (Lesch et al., 1996; Ferrari et al., 2005; Frankle et al., 2005; Canli and Lesch, 2007; Coccaro et al., 2010). The short 5-HTTPLR variant is associated with a lower transcriptional activity leading to a reduced 5-HTT production (Canli and Lesch, 2007). Carriers of one or two copies of the short 5-HTTLPR variant have higher scores of neuroticism, a trait related to anxiety and depression (Canli and Lesch, 2007). Also, gene-by-environment interactions have been reported, showing that individuals with two copies of the short variant have an increased risk of developing depression after experiencing stressful life-events (Caspi et al., 2003; Reif et al., 2007; Sugden et al., 2010; Karg et al., 2011). To investigate the effects of varying levels of the 5-HTT in rodents, a 5-HTT knockout mouse model with a targeted disruption of the 5-HTT gene was developed. Similar to findings in humans and primates, total and partial inactivation of the gene is related to increased levels of anxiety-like behavior (Holmes et al., 2003; Carroll et al., 2007; Heiming and Sachser, 2010; Araragi and Lesch, 2013) and a decrease in aggressiveness (Holmes et al., 2002; Lewejohann et al., 2010; Jansen et al., 2011; Heiming et al., 2013; but see Kloke et al., 2011).

The aim of the present study was to investigate how serotonin transporter genotype and social experiences during adolescence affect anxiety-like and aggressive behavior in male mice. For this purpose, male 5-HTT knockout mice and their wildtype counterparts experienced either social adversity or beneficial conditions throughout adolescence and afterwards were tested for anxiety-like and aggressive behavior in a battery of standard behavioral tests. We hypothesized that animals that experienced adversity differ in their levels of anxiety-like behavior (i) and aggressiveness (ii) compared to animals that experienced beneficial conditions. Concerning effects of genotype, we hypothesized that mice with lower levels of the serotonin transporter show higher levels of anxiety-like behavior (iii) and lower levels of aggressiveness (iv).

## Methods

### Animals and housing conditions

The present study was performed with male heterozygous (+∕−) and homozygous (−∕−) serotonin transporter (5-HTT) knockout mice as well as their wildtype (+∕+) littermates (Bengel et al., 1998). They originated from our local breeding stock (Department of Behavioral Biology, University of Münster, Germany) consisting of heterozygous breeding pairs; the original stock was contributed by the Department of Molecular Psychiatry, University of Würzburg, Germany. Mice were genotyped by extracting genomic DNA from ear tissue and amplifying it by PCR. Genotypes were identified via agarose gel electrophoresis of DNA fragments of either 225 bp (5-HTT +∕+), 272 bp (5-HTT −∕−), or both (5-HTT+∕−).

Litters were not culled. Litter sizes varied from 3 to 12 offspring. With one exception only mixed-sex litters were used. Male mice were weaned on postnatal day (PND) 21 ± 1 and housed in same-sex but mixed-genotype groups of two to five siblings until the start of the experiment. Both, breeding pairs and same-sex groups were housed in standard Macrolon cages type III (37 cm × 21 cm × 15 cm) equipped with sawdust (Allspan, Höveler, Langenfeld, Germany) as bedding material, a paper towel as nesting material and structural enrichment (plastic housing and wooden scaffolding or stick). Water and food (1324, Altromin GmbH, Lage, Germany) were provided *ad libitum*. Housing rooms were maintained at a 12 h dark/light cycle, a temperature of 22 ± 2°C and a relative air humidity of 50 ± 10%. In total 78 mice (26 5-HTT +∕+, 27 5-HTT +∕−, 25 5-HTT −∕−) were used for behavioral and endocrine investigations. Deviations from sample sizes are due to technical reasons and the exact sample sizes are given in the Results section.

All procedures complied with the regulations covering animal experimentation within the EU (European Communities Council DIRECTIVE 2010/63/EU). They were conducted in accordance with the institution's animal care and use guidelines and approved by the national and local authorities (LANUV-NRW; reference number: 84-02.05.20.12.099).

### Experimental design

**Figure 1:**
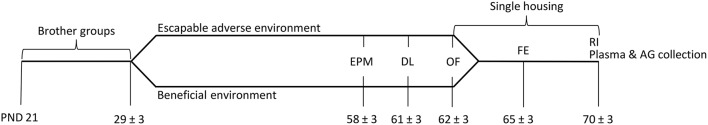
**Timeline of experimental design**. After weaning at postnatal day (PND) 21, experimental animals lived in brother groups. Starting on PND 29 ± 3, experimental mice either experienced an escapable adverse or a beneficial environment. To assess anxiety-like behavior, the Elevated Plus Maze test (EPM), Dark-Light test (DL), and Open Field test (OF) were conducted. After the OF, the animals were housed singly. On PND 65 ± 3 the Free Exploration test (FE) was performed and on PND 70 ± 3 aggressiveness was determined in a Resident Intruder test (RI). Trunk blood was collected to investigate plasma corticosterone and testosterone levels and adrenal glands (AG) were dissected to determine tyrosine hydroxylase activity.

The aim of the present study was to investigate how either a beneficial environment or escapable social adversity that male 5-HTT +∕+, 5-HTT +∕− and 5-HTT −∕− mice experience during adolescence affect their anxiety-like behavior and aggressiveness. For this purpose, two different environments were created that are described in more detail below. Additionally, endocrinological parameters (plasma corticosterone and testosterone, adrenal tyrosine hydroxylase) were assessed. A timeline of the experiment is shown in Figure 1.

#### Creating different social experiences

**Figure 2:**
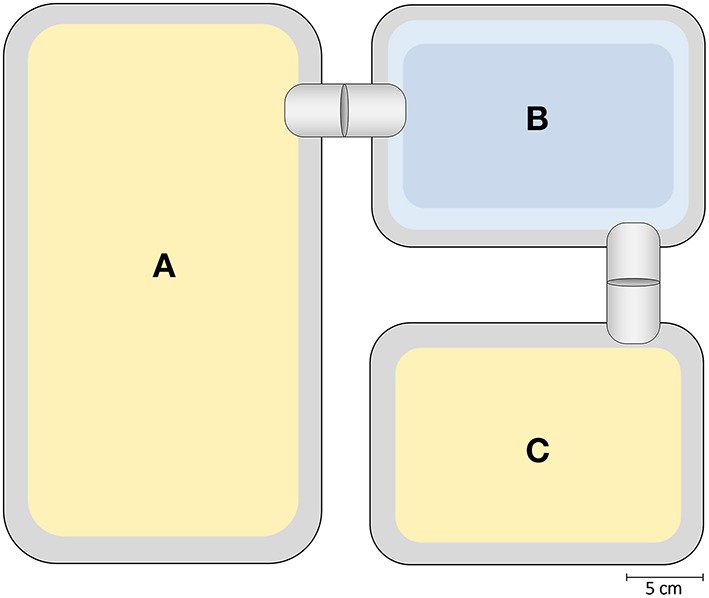
**Schematic overview of custom made cage system**. The **“**Interaction cage” **(A)** is connected to the “Refuge cage” **(C)** via a water pool **(B)**. Cages are connected with plastic tubes that can be sealed by inserting a PVC plate into an incision. The “Interaction cage” and “Refuge cage” are filled with sawdust; food and water is provided *ad libitum*. The water basin is filled to a height of 2 – 2.5 cm.

Two experimental groups were established, based on the social experience during adolescence: While one group experienced an escapable adverse social environment (AE), the other group experienced a beneficial social environment (BE) (PND 29 ± 3 − 62 ± 3). AE animals experienced repeated aggression from which they could escape by crossing a water basin, while BE animals had free access to a female mating partner. For this purpose, animals were housed in a custom-made cage system (first described in Lewejohann et al., 2010), which consisted of a standard Macrolon cage type III (“Interaction cage”) that was connected to a Macrolon cage type II (22 cm × 16 cm × 14 cm, “Refuge cage”) via plastic tubes (Ø 3.5 cm) and a water pool (modified Macrolon cage type II; Figure 2). Both housing cages were filled with sawdust as bedding material and provided with a paper towel as nesting material. The tubes could be sealed by inserting a PVC plate into an incision in the middle of the plastic tube. The basin was filled with water to a height of approximately 2.0–2.5 cm and equipped with a wire mesh grid on the ground. Mice would not voluntarily cross the water basin, but if attacked by a dominant animal, they would use it to escape.

#### Habituation

Twelve hours before the individual animals were transferred to the different environments, they were housed in the cage systems in their sibling groups to familiarize with the cage system; during this time the bottom of the water-basin was only slightly moist and the tunnel leading to the smaller cage was baited with oat-flakes.

#### Adverse social experience (AE)

In the interaction cage, an established couple was housed, consisting of a female mouse from the NMRI strain and a male mouse from the CD-1 strain [both strains obtained from Harlan Laboratories (Venray, The Netherlands)]. The CD-1 strain was chosen to ensure that the 5-HTT mice encounter a larger and dominant opponent because CD-1 mice are generally heavier than 5-HTT mice. To avoid a bias that might have been caused by female choice for a mate of the same strain, a female from another strain (NMRI) was chosen. On PND 29 ± 3 the experimental animal was introduced to the “Interaction cage” for the first time. Sessions lasted 3.5 h or until the animals crossed the water basin. If escalated aggression (see Table 1) occurred, the session was terminated prematurely by moving the experimental animal to the refuge cage. After the sessions the connection tubes were sealed with a PVC plate. This procedure was repeated 5 days a week during the first 3 h of the light phase.

**Table 1:** **Definition of different behaviors in the Resident Intruder test**.

**Behavior**	**Definition**	**Measure**
**SOCIAL INTEREST BEHAVIOR**		
Approach	Directed movement toward the other mouse at a walking or running pace until the distance between both mice is at most one body length.	Number
Nasal sniffing	The mouse contacts the nasal region of a conspecific with the twitching tip of its snout.	Duration
Anogenital sniffing	The mouse contacts the anogenital region of a conspecific with the twitching tip of its snout.	Duration
Following	The mouse runs after the other mouse, while the head of the following mouse is directed at the backside of the other individual. The maximum distance between the animals is one body length. After stopping forward motion for at least 3 s, additional following is considered a new event.	Duration
**AGONISTIC BEHAVIOR**		
Attack	A mouse contacts the body of the other mouse with its mouth, making that mouse react with winced movements of single extremities, the tail, or the whole body. Attacks are single countable events of low intensity.	Number
Attack latency	Time that elapses until attacking is performed for the first time by the focal animal. If no attacking occurs, the latency is set to the maximal testing time of 10 min.	Duration
Sustained attack	A series of attacks with rushing and leaping of the other mouse. As the behavior is of higher intensity than the attack itself, single attacks are not discriminable.	Number
Escalated fighting	Physical struggle between two mice, initiated by an attack and usually involving further attacks, kicking, wrestling, and rolling in the bedding.	Number
Chasing	‘Following’ subsequent to an agonistic interaction (attacking, sustained attacking, or escalated fighting).	Duration

*For description of behavior patterns see also (Marashi et al., 2003, 2004; Jansen et al., 2010, 2011; Kloke et al., 2011)*.

#### Beneficial social experience (BE)

A female mouse from the NMRI strain was housed in the main cage and the focal animal was introduced for the first time on PND 29 ± 3. To resemble the handling of the AE condition, BE mice were taken out and re-introduced to the main cage 5 days per week in the first 3 h of the light period and connection tubes were opened. After the session ended, the focal animal stayed with the female and connection tubes were sealed again. All experimental animals were weighed weekly.

### Behavioral testing

To test state anxiety [i.e., the anxiety experienced at a certain moment that can be augmented by anxiogenic stimuli (Lister, 1990; Griebel et al., 1993)] of all experimental animals, the Elevated Plus Maze test (EPM; PND 58 ± 3), Dark-Light test (DL; PND 61 ± 3), and Open Field test (OF; PND 62 ± 3) were performed while the animals were still housed in the cage-system. The tests were always conducted in the same order and at equal between-test intervals. The animals were tested in the first 5 h of the light period and at least 45 min after the successful escape from the main cage. In order to test for trait anxiety [i.e., the general tendency to display anxious behavior without the presence of a threat (Griebel et al., 1993)] in the Free Exploration test (FE; PND 65 ± 3), the animals were housed singly for 3 days (in a modified Macrolon cage type III that was equipped with a sliding door on one of the short sides). In contrast to the EPM, DL, and OF, where the animals are exposed to the different apparatuses, they can freely choose to explore an unknown arena in the FE (Griebel et al., 1993).

#### Elevated plus maze test

The EPM consisted of a gray plastic apparatus with four arms set in a cross from a central square (5 cm × 5 cm). Two opposing arms were delimited by vertical walls (20 cm high) (closed arms), whereas the other two arms were surrounded by a small border (5 mm) to prevent the mice from falling off (open arms). The maze was elevated 50 cm from the ground and illumination level was 150 lx in the central square. After spending 1 min in an empty Macrolon cage type II, each mouse was placed in the central zone, always facing toward the same closed arm, and was allowed to investigate the EPM for 5 min. The mouse was tracked using the program ANY-maze (Stoelting, IL, USA). Relative entries into open arms (open arm entries divided by sum of open and closed arm entries), relative time spent on open arms (time on open arms divided by time on open and closed arms), distance on open arms and total distance traveled were measured.

#### Dark-light test

The DL test apparatus consisted of a modified Macrolon cage type III, which was divided into two compartments by a gray PVC plate. The dark compartment comprised one third of the cage, was painted black and could be covered with a dark lid. The light compartment consisted of two thirds of the cage with transparent walls, and was illuminated from above (570 lx). The two compartments were connected via a small sliding door (4 cm × 10 cm) inserted in the PVC plate. Each mouse was placed inside the dark compartment with lid and sliding door closed. The individual remained there for 1 min before the sliding door was opened and the mouse could freely explore the DL apparatus for 5 min. The mouse was tracked using the program ANY-maze. The parameters analyzed for each animal were the latency to enter the light compartment, the number of entries into the light compartment and the time spent in the light compartment.

#### Open field test

The OF test apparatus was a white 80 cm × 80 cm x 40 cm arena surrounded by white walls that was illuminated by an overhead bulb (600 lx). The center zone was defined as an area of 40 cm × 40 cm in the middle of the OF. After spending 1 min in a dark cylinder that was placed in one corner, the cylinder was lifted and the individual was allowed to freely explore the OF for 5 min. The mouse was tracked using the program ANY-maze. The parameters analyzed were the path length, the number of center entries and the time spent in the center zone.

#### Free exploration test

The apparatus of the FE was a white 60 cm × 60 cm × 35 cm arena surrounded by white walls that was illuminated from the top (180 lx). An opening in one wall (11 cm × 15 cm) connected to the home cage of the animal via a Plexiglas tunnel. After spending 1 min in an empty Macrolon cage type II, the mice were placed back into their home cage and a sliding door was opened, allowing the individual to explore the FE arena for 15 min. The tests were recorded and analyzed using the software Optimas 6.5N (Media Cybernetics) and Tracking Analysis 1.1.1 (Lars Lewejohann, www.phenotyping.com). Latency to enter the arena, the entries and time in the arena and the total distance traveled in the arena were analyzed.

#### Resident intruder paradigm (RI)

The focal animal stayed in its home cage and a male of the docile C3H strain (obtained from Harlan Laboratories (Venray, The Netherlands)) was introduced as an intruder. The encounter was recorded for 10 min; in one case the confrontation had to be stopped prematurely to prevent injury of the C3H (data were excluded from analysis). The behavior of the animals was recorded using the software Observer XT 8.0 (Noldus Information Technology, Wageningen, The Netherlands). The experimenter remained blind to the genotypes and the social experiences of the animals. For definitions of the recorded behaviors see Table 1.

### Analysis of endocrine parameters

#### Plasma corticosterone and testosterone levels

##### Blood sampling

Ten minutes after the RI (PND 70 ± 3), the animals were anesthetized using the inhalation anesthetic Isoflurane (Forene, Abbott GmbH, Wiesbaden, Germany). Once the mouse reached deep sedation it was decapitated and trunk blood was collected in heparinised capillaries within 3 min of catching the animal. After separation of cellular constitutions by centrifugation (5 min at 13.000 rpm), plasma was frozen at −20°C until analysis.

##### Hormone analysis

For the analysis of plasma corticosterone and testosterone concentrations, blood samples were analyzed using an established Demeditec Enzyme Immunoassay Kit (EIA, DE4164 and EIA, DES6622, Demeditec Diagnostics GmbH, Kiel, Germany, respectively). All standards, samples, and controls were run in duplicate concurrently. Intra- and inter-assay coefficients of variation were below 10 and 12%, respectively (see Jansen et al., 2011).

#### Tyrosine hydroxylase activity

Adrenal glands were collected within 10 min of decapitation and fast frozen in a Tris-HCl buffer (pH 7.2) on dry ice. For analysis of the tyrosine hydroxylase (TH) activity, the adrenals were gently defrosted and homogenized in 150 μl 5 mM Tris–HCl buffer (pH 7.2). After centrifugation (14.000 rpm) for 30 min at 4°C, TH was determined in the supernatant by means of a radioenzymatic method according to the method of Nagatsu et al. (1964) with slight modifications as described in Witte and Matthaei (1980), see also Kaiser and Sachser (1998) and Jansen et al. (2011).

### Statistical analysis

Residuals were tested for normal distribution. When necessary, raw data were transformed using either square-root- or ln-transformation (Square-root: EPM: distance on open arm, OF: center time; Logarithmic: OF: latency to enter center, time in center, DL: time in light compartment, latency to enter light compartment). Data were analyzed using analysis of variance (ANOVA) with “environment” and “genotype” as fixed factors and followed by Tukey HSD post-hoc testing. In case of repeated measures (body weight), a repeated-measures ANOVA (RM ANOVA) was carried out. Because not all data of the RI could be transformed, all RI parameters were analyzed non-parametrically using Mann-Whitney U for social experience and Kruskal-Wallis for genotype [in case of significance, pairwise comparisons between genotypes were subsequently performed using Mann–Whitney U test (Bonferroni corrected)]. The same holds true for testosterone data. Data were analyzed using IBM SPSS Statistics for Windows, Version 22.0, released 2013. Graphs were created with SigmaPlot Version 12.5 (Build 12.5.0.83, Systat Software, Inc. 2011). Data are presented as bars with means and standard error of the mean (SEM) or box-plots with medians, 25–75% quartiles and 10–90% ranges. Differences of *p* ≤ 0.05 were considered significant, *p*-values ranging from > 0.05 to < 0.1 were regarded as trends.

## Results

### Effects of social experience and genotype on body weight gain

**Figure 3:**
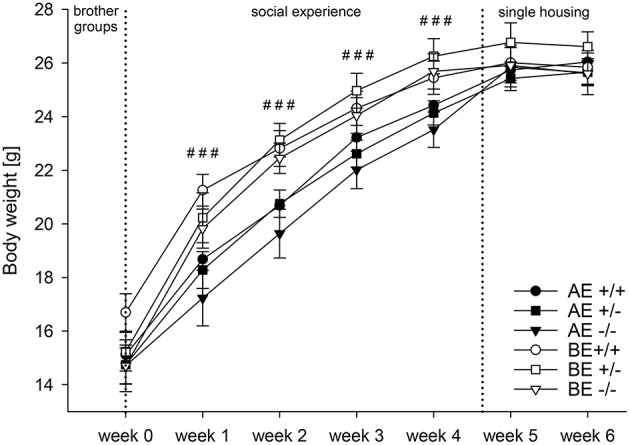
**Body weight (in gram) measured weekly of male heterozygous (+∕−) and homozygous (−∕−) 5-HTT knockout mice and their wildtype counterparts (+∕+) that experienced either an adverse (AE, closed symbols) or beneficial (BE, open symbols) environment during adolescence (AE: 5-HTT +∕+ n = 12, 5-HTT +∕− n = 13, 5-HTT −∕− n = 13; BE: 5-HTT +∕+ n = 13, 5-HTT +∕− n = 13, 5-HTT −∕− n = 11)**. Values are presented as group means ± SEM. Univariate ANOVA: Effect of environment: ^###^ *p* ≤ 0.001. Different social experiences started in week 0 (dashed line) and lasted until week 4 (dashed line). Hereafter, animals were singly housed and no longer experienced different environments.

Univariate ANOVA detected that body weight did not differ significantly between AE and BE animals or between genotypes before the start of the experiment [week 0: social experience: *F*_(1, 69)_ = 1.234, *p* = 0.271; genotype: *F*_(2, 69)_ = 0.847, *p* = 0.433]. Repeated measures ANOVA revealed a main effect of time [*F*_(2.926, 201.898)_ = 873.852, *p* < 0.001; Greenhouse Geisser corrected; Figure 3]. All groups gained weight over the course of the experiment. Moreover, a main effect of social experience was detected [*F*_(1, 69)_ = 8.106, *p* = 0.006] and an interaction between time and social experience was found [*F*_(2.926, 201.898)_ = 11.661, *p* < 0.001; Greenhouse Geisser corrected]. AE mice were markedly lighter than BE mice [week 1: *F*_(1, 69)_ = 14.775, *p* < 0.001; week 2: *F*_(1, 69)_ = 17.572, *p* < 0.001; week 3: *F*_(1, 69)_ = 12.617, *p* = 0.001; week 4: *F*_(1, 69)_ = 12.555, *p* = 0.001]. Two days after the animals were housed singly, no differences between AE and BE mice were found anymore [week 5: *F*_(1, 69)_ = 1.179, *p* = 0.281; week 6: *F*_(1, 69)_ = 0.170; *p* = 0.681]. No main effect of genotype [*F*_(2, 69)_ = 0.411, *p* = 0.655] and no interaction between time and genotype were detected [*F*_(5.852, 201.898)_ = 1.717, *p* = 0.120; Greenhouse Geisser corrected; week 1–week 6: *F*_(2, 69)_ = 0.363, *F*_(2, 69)_ = 0.831, *p* = 0.447; week 2; *p* = 0.440, *p* = 0.697]. No interaction between social experience and genotype interaction was observed [*F*_(2, 69)_ = 0.093, *p* = 0.912].

### Effects of social experience and genotype on anxiety-like and exploratory behavior

#### Effects of social experience on anxiety-like and exploratory behavior

**Figure 4:**
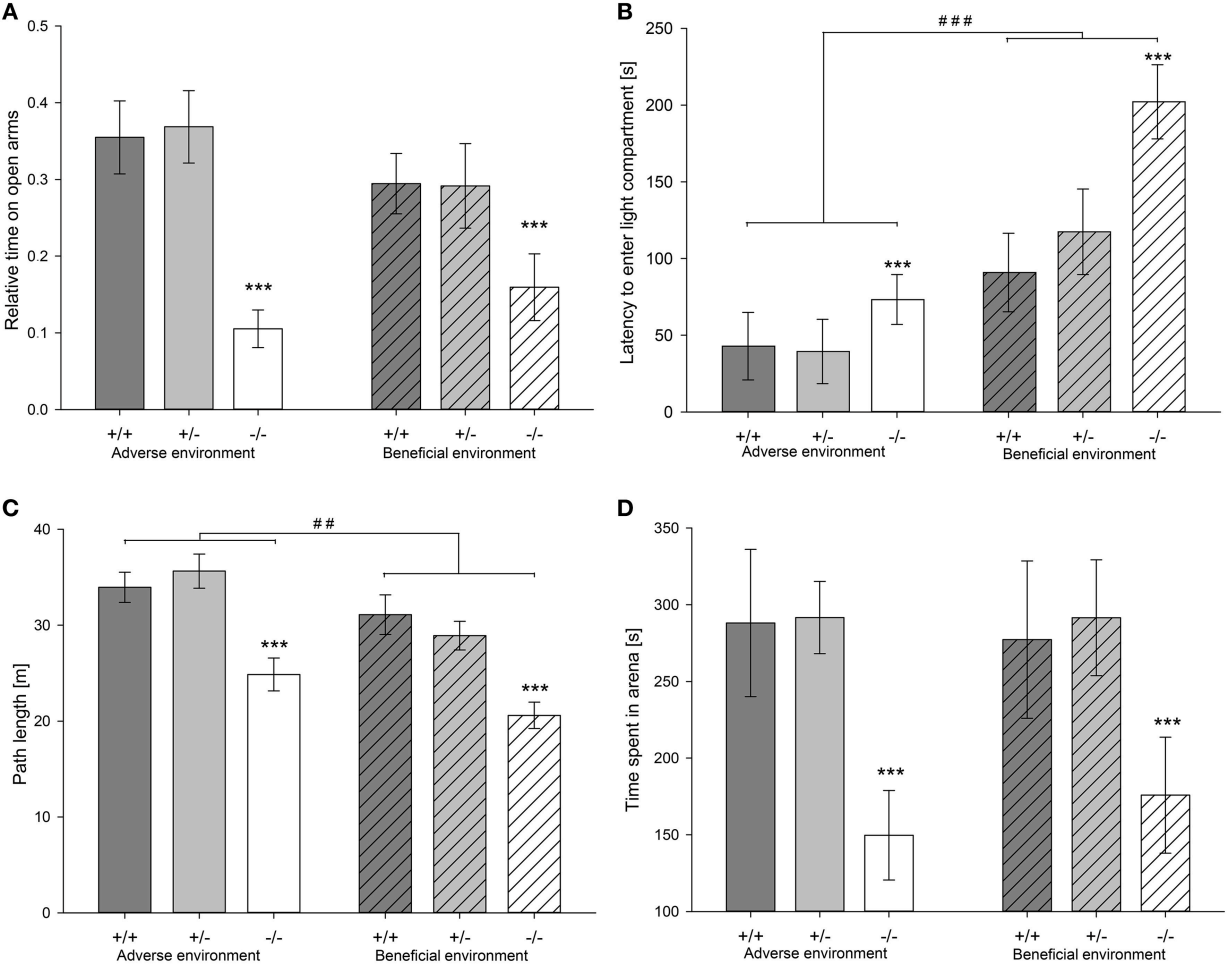
**Anxiety-like behavior of male heterozygous (+∕−) and homozygous 5-HTT knockout (−∕−) mice and their wildtype counterparts (+∕+) that experienced either an adverse or beneficial environment during adolescence. (A)** Relative time spent on the open arms in the EPM (AE: 5-HTT +∕+ n = 13, 5-HTT +∕− n = 14, 5-HTT −∕− n = 13; BE: 5-HTT +∕+ n = 13, 5-HTT +∕− n = 13, 5-HTT −∕− n = 12). **(B)** Latency (s) to enter the lit compartment in the DL (AE: 5-HTT +∕+ n = 12, 5-HTT +∕− n = 13, 5-HTT −∕− n = 13; BE: 5-HTT +∕+ n = 13, 5-HTT +∕− n = 13, 5-HTT −∕− n = 11). **(C)** Distance traveled (m) in the OF (AE: 5-HTT +∕+ n = 12, 5-HTT +∕− n = 13, 5-HTT −∕− n = 13; BE: 5-HTT +∕+ n = 13, 5-HTT +∕− n = 13, 5-HTT −∕− n = 11). **(D)** Time (s) spent in the arena of the FE (AE: 5-HTT +∕+ n = 12, 5-HTT +∕− n = 13, 5-HTT −∕− n = 13; BE: 5-HTT +∕+ n = 13, 5-HTT +∕− n = 13, 5-HTT −∕− n = 11). Values are presented as group means ± SEM. Effect of genotype: ^***^p ≤ 0.001; effect of social experience: ^##^*p* ≤ 0.01, ^###^*p* ≤ 0.001.

While there was no effect of social experience on measures of anxiety-like behavior in the EPM (Figure 4A; Table 2), explorative locomotion measured as total distance traveled in the apparatus was significantly increased by adverse experiences [*F*_(1, 72)_ = 10.966, *p* = 0.001] (Table 2). In the DL, there was a significant main effect of social experience, with animals from the adverse condition displaying significantly less anxiety-like and more exploratory behavior compared to animals that experienced beneficial conditions: AE animals had a shorter latency to enter the light compartment [*F*_(1, 72)_ = 17.076, *p* < 0.001; Figure 4B, Table 2], entered it more often [*F*_(1, 72)_ = 12.824, *p* = 0.001] and furthermore showed a trend to spend more time in it [*F*_(1, 72)_ = 2.824, *p* = 0.097; Table 2]. There was a significant main effect of social experience on anxiety-like behavior in the OF. AE animals entered the center more often than BE mice [*F*_(1, 72)_ = 4.979, *p* = 0.029]. Besides, there was a trend that AE animals spent more time in the center zone than BE mice [*F*_(1, 72)_ = 3.856, *p* = 0.053; Table 2]. Moreover, exploratory behavior, measured as total distance traveled in the OF, was significantly influenced by social experience [*F*_(2, 72)_ = 9.815, *p* = 0.003]. AE animals covered a greater distance compared to BE animals (Figure 4C, Table 2). In the FE, social experience affected neither anxiety-like nor exploratory behavior measured by latency to enter the arena [*F*_(1, 63)_ = 2.552; *p* = 0.155], number of entries to the arena [*F*_(1, 63)_ = 0.212; *p* = 0.647], time spent in the arena [*F*_(1, 63)_ < 0.001; *p* = 0.990; Figure 4D] and total distance traveled [*F*_(1, 63)_ = 0.078; *p* = 0.781; Table 2].

**Table 2:** **Group means ± SEM of anxiety-like behavior and exploratory locomotion of male heterozygous (+∕−) and homozygous (−∕−) 5-HTT knockout mice and their wildtype counterparts (+∕+) that experienced either an adverse (AE) or beneficial (BE) environment during adolescence**.

**Anxiety-like behavior and exploratory locomotion Parameter**	**AE**			**BE**			**Effect of environment (E)**	**Effect of genotype (G)**	**G × E**
	**+∕+**	**+∕−**	**−∕−**	**+∕+**	**+∕−**	**−∕−**			
**ELEVATED PLUS MAZE**									
Open arm entries (relative)	0.44 ± 0.04	0.42 ± 0.04	0.23 ± 0.04	0.41 ± 0.03	0.41 ± 0.05	0.35 ± 0.06	*F*_(1, 72)_ = 0.634; *p* = 0.429	***F*_(2, 72)_ = 6.143; *p* = 0.003**	*F*_(2, 72)_ = 1.681; *p* = 0.193
Time on open arms (relative)	0.35 ± 0.05	0.37 ± 0.05	0.11 ± 0.02	0.29 ± 0.04	0.29 ± 0.06	0.16 ± 0.04	*F*_(1, 72)_ = 0.591; *p* = 0.445	***F*_(2, 72)_ = 12.805; *p* < 0.001**	*F*_(2, 72)_ = 1.289; *p* = 0.282
Distance on open arms (m)	3.09 ± 0.51	2.79 ± 0.43	0.61 ± 0.19	2.29 ± 0.47	2.14 ± 0.41	1.23 ± 0.38	*F*_(1, 72)_ = 0.318; *p* = 0.575	***F*_(2, 72)_ = 16.153; *p* < 0.001**	*F*_(2, 72)_ = 1.840; *p* = 0.166
Total distance (m)	11.45 ± 0.72	10.33 ± 0.72	7.99 ± 0.67	9.70 ± 0.72	8.93 ± 0.61	5.56 ± 0.66	***F*_(1, 72)_ = 10.966; *p* = 0.001**	***F*_(2, 72)_ = 16.104; *p* < 0.001**	*F*_(2, 72)_ = 0.286; *p* = 0.752
**DARK-LIGHT**									
Entries to light compartment (#)	11.92 ± 1.78	10.57 ± 1.82	6.46 ± 0.96	7.57 ± 1.11	6.93 ± 1.17	2.83 ± 0.71	***F*_(1, 72)_ = 12.824; *p* = 0.001**	***F*_(2, 72)_ = 7.577; *p* = 0.001**	*F*_(2, 72)_ = 0.027; *p* = 0.974
Time in light compartment (s)	77.18 ± 12.14	72.59 ± 11.56	32.32 ± 5.30	58.25 ± 14.53	63.62 ± 17.18	19.26 ± 6.34	*F*_(1, 72)_ = 2.824; *p* = 0.097	***F*_(2, 72)_ = 7.664; *p* = 0.001**	*F*_(2, 72)_ = 0.031; *p* = 0.970
Latency to enter light compartment (s)	45.85 ± 23.59	42.21 ± 22.30	73.23 ± 16.26	94.34 ± 23.96	109.07 ± 27.13	202.23 ± 24.20	***F*_(1, 72)_ = 17.076; *p* < 0.001**	***F*_(2, 72)_ = 7.156; *p* = 0.001**	*F*_(2, 72)_ = 0.202; *p* = 0.818
**OPEN FIELD**									
Total distance (m)	33.63 ± 1.66	35.43 ± 1.90	24.87 ± 1.72	31.10 ± 2.10	28.92 ± 1.50	20.60 ± 1.38	***F*_(1, 72)_ = 9.815; *p* = 0.003**	***F*_(2, 72)_ = 19.756; *p* < 0.001**	*F*_(2, 72)_ = 0.677; *p* = 0.511
Center entries (#)	10.00 ± 1.21	10.00 ± 0.96	4.46 ± 0.87	7.54 ± 1.31	7.62 ± 1.30	3.33 ± 0.74	***F*_(1, 72)_ = 4.979; *p* = 0.029**	***F*_(2, 72)_ = 13.092; *p* < 0.001**	*F*_(2, 72)_ = 0.230; *p* = 0.795
Time in center (s)	16.15 ± 2.71	16.97 ± 2.38	7.39 ± 1.44	11.12 ± 1.85	13.13 ± 2.90	5.82 ± 1.27	*F*_(1, 72)_ = 3.856; *p* = 0.053	***F*_(2, 72)_ = 10.900; *p* < 0.001**	*F*_(2, 72)_ = 0.263; *p* = 0.769
**FREE EXPLORATION**									
Latency to enter arena (s)	129.22 ± 51.81	111.06 ± 21.76	351.83 ± 72.06	161.25 ± 84.92	88.71 ± 21.66	251.31 ± 73.03	*F*_(1, 63)_ = 2.552; *p* = 0.115	***F*_(2, 63)_ = 12.377; *p* < 0.001**	*F*_(2, 63)_ = 0.063; *p* = 0.939
Entries to arena (#)	30.00 ± 3.39	33.08 ± 2.55	19.67 ± 2.33	29.8 ± 4.11	26.00 ± 2.04	21.27 ± 4.84	*F*_(1, 63)_ = 0.212; *p* = 0.647	***F*_(2, 63)_ = 6.617; *p* = 0.002**	*F*_(2, 63)_ = 1.454; *p* = 0.241
Total distance (m)	30.90 ± 4.19	27.74 ± 2.67	14.54 ± 2.90	27.58 ± 5.05	26.42 ± 3.26	16.72 ± 3.60	*F*_(1, 63)_ = 0.078; *p* = 0.781	***F*_(2, 63)_ = 8.144; *p* = 0.001**	*F*_(2, 63)_ = 0.288; *p* = 0.751
Time in arena (s)	313.34 ± 44.74	280.59 ± 22.47	149.72 ± 29.16	277.29 ± 51.25	291.55 ± 37.76	175.89 ± 37.85	*F*_(1, 63)_ < 0.001; *p* = 0.990	***F*_(2, 63)_ = 7.919; *p* = 0.001**	*F*_(2, 63)_ = 0.363; *p* = 0.697

*Effects of environment, genotype and gene by environment interaction (G x E) on anxiety-like behavior and exploratory locomotion. Statistics: ANOVA, bold: p ≤ 0.05. EPM, DL, OF: AE +∕+ n = 13, AE +∕− n = 14, AE −∕− n = 13, BE +∕+ n = 13, BE +∕− n = 13, BE −∕− n = 12; FE: AE +∕+ n = 11, AE +∕− n = 13, AE −∕− n = 12, BE +∕+ n = 10, BE +∕− n = 12, BE −∕− n = 11*.

#### Effects of 5-HTT genotype on anxiety-like and exploratory behavior

Genotype significantly affected both anxiety-like behavior and explorative locomotion in the EPM. 5-HTT −∕− mice showed fewer open arm entries, spent less time on the open arms and traveled a shorter distance on the apparatus than 5-HTT +∕+ and 5-HTT +∕+ animals [relative open arm entries: *F*_(2, 72)_ = 6.143, *p* = 0.003, Tukey HSD: 5-HTT +∕+ vs. 5-HTT −∕−: *p* = 0.006, 5-HTT +∕− vs. 5-HTT −∕−: p = 0.010; relative time on open arms: *F*_(2, 72)_ = 12.805, *p* < 0.001, Tukey HSD: 5-HTT +∕+ vs. 5-HTT −∕−: *p* < 0.001, 5-HTT +∕− vs. 5-HTT −∕−: *p* < 0.001; total distance: *F*_(2, 72)_ = 16.104, *p* < 0.001, Tukey HSD: 5-HTT +∕+ vs. 5-HTT −∕−: *p* < 0.001, 5-HTT +∕− vs. 5-HTT −∕−: *p* < 0.001; Figure 4A, Table 2]. Similarly, a significant main effect of genotype on anxiety-like and exploratory behavior could be detected in the DL [latency to enter lit compartment: *F*_(2, 72)_ = 7.156, *p* = 0.001; entries to lit compartment: *F*_(2, 72)_ = 7.577, *p* = 0.001; time in lit compartment: *F*_(2, 72)_ = 7.664, *p* = 0.001]. 5-HTT −∕− mice showed significantly higher levels of anxiety-like behavior than 5-HTT +∕+ (Tukey HSD; number of entries: *p* = 0.001; time in lit compartment: *p* = 0.002, latency: *p* = 0.004) and 5-HTT +∕− mice (Tukey HSD; number of entries: *p* = 0.016; time in lit compartment: *p* = 0.006, latency: *p* = 0.007; Figure 4B). Genotype significantly affected behavior in the OF. Genotypes differed in the number of center entries [*F*_(2, 72)_ = 13.092, *p* < 0.001] and time spent in the center [*F*_(2, 72)_ = 10.900, *p* < 0.001]: 5-HTT −∕− mice entered the center fewer times and spent less time in it than 5-HTT +∕+ (Tukey HSD; entries: *p* < 0.001, time: *p* < 0.001) and 5-HTT +∕− animals (Tukey HSD; entries: *p* < 0.001, time: *p* < 0.001). Additionally, total distance traveled was affected by genotype [*F*_(2, 72)_ = 19.756, *p* < 0.001]. 5-HTT −∕− mice traveled less than 5-HTT +∕+ (Tukey HSD; *p* < 0.001) and 5-HTT +∕− mice (Tukey HSD; *p* < 0.001; Figure 4C). In contrast to the effect of experience, genotype significantly influenced anxiety-like and exploratory behavior in the FE [latency to enter arena: *F*_(2, 63)_ = 12.377, *p* < 0.001; number of entries: *F*_(2, 63)_ = 6.617, *p* = 0.002; time in arena: *F*_(2, 63)_ = 7.919, *p* = 0.001; path length: *F*_(2, 63)_ = 8.144, *p* = 0.001) with 5-HTT −∕− mice having a longer latency to enter the arena (5-HTT −∕− vs. 5-HTT +∕+: *p* < 0.001 and 5-HTT −∕− vs. 5-HTT +∕−: *p* < 0.001 (Tukey HSD)], entering the arena less often [5-HTT −∕− vs. 5-HTT +∕+: *p* = 0.007 and 5-HTT −∕− vs. 5-HTT +∕−: *p* = 0.005 (Tukey HSD)], spending less time in the arena [5-HTT −∕− vs. 5-HTT +∕+: *p* = 0.002 and 5-HTT −∕− vs. 5-HTT +∕−: *p* = 0.003 (Tukey HSD)] and traveling a shorter distance [5-HTT −∕− vs. 5-HTT +∕+: *p* = 0.001 and 5-HTT −∕− vs. 5-HTT +∕−: *p* = 0.005 (Tukey HSD)] compared to 5-HTT +∕+ and 5-HTT +∕− mice (Figure 4D).

No interaction between social experience and genotype were detected for anxiety-like behavior (Table 2).

### Effects of social experience and genotype on agonistic behavior

The Resident Intruder test took place in the home cage of the focal animal. In the beginning of the encounter, the animals mainly showed social interest behaviors like approaching or sniffing or the animals didn't interact with each other. On average after 5 min, agonistic interactions started, however, social interest behavior was still observed.

#### Effects of social experience on agonistic behavior

**Figure 5:**
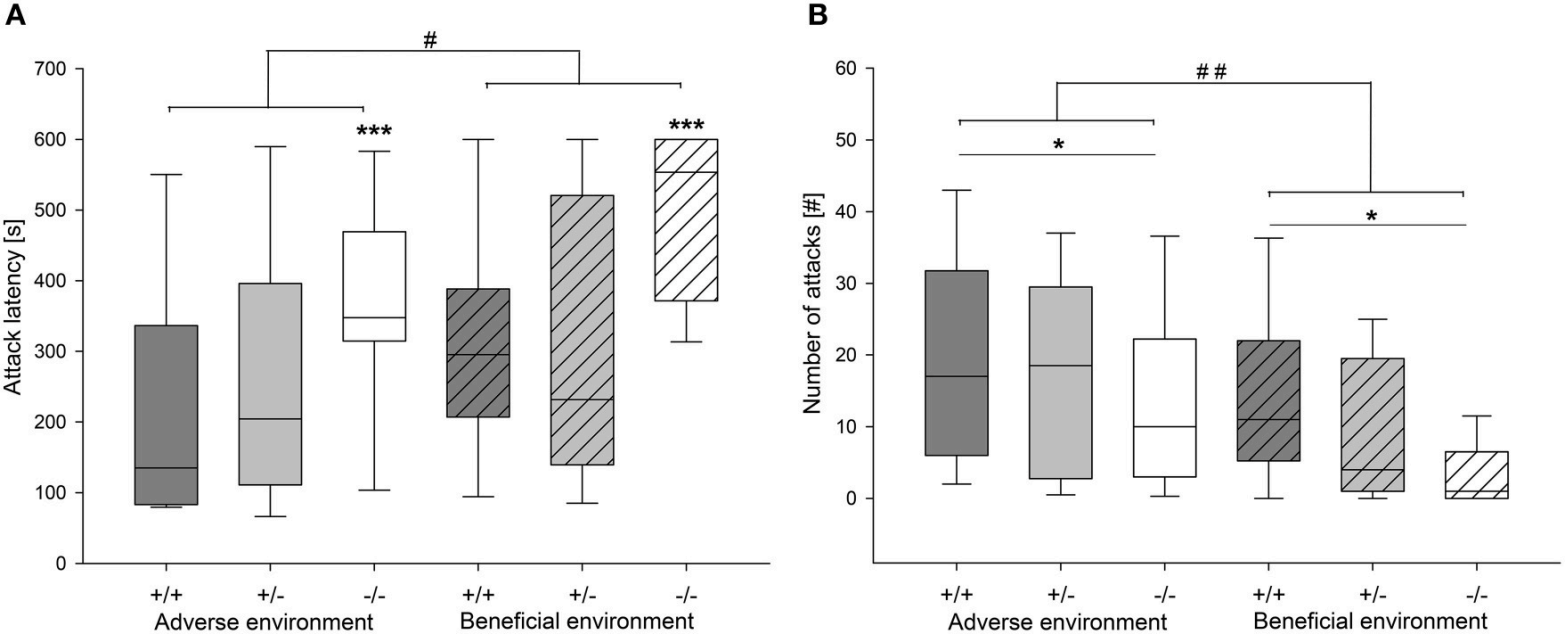
**Agonistic behavior in the RI test of male heterozygous (+∕−) and homozygous 5-HTT knockout (−∕−) mice and their wildtype counterparts (+∕+) that experienced either an adverse or beneficial environment during adolescence. (A)** attack latency in seconds; **(B)** number of attacks. Values are presented as box plots with medians, 25–75% quartiles and 10–90% ranges. Effect of social experience: ^#^*p* ≤ 0.05, ^##^*p* ≤ 0.01; effect of genotype ^*^p ≤ 0.05, ^***^p ≤ 0.001; AE +∕+ n = 13, AE +∕− n = 13, AE −/− n = 12, BE +∕+ n = 12, BE +∕− n = 13, BE −/− n = 12.

**Figure 6:**
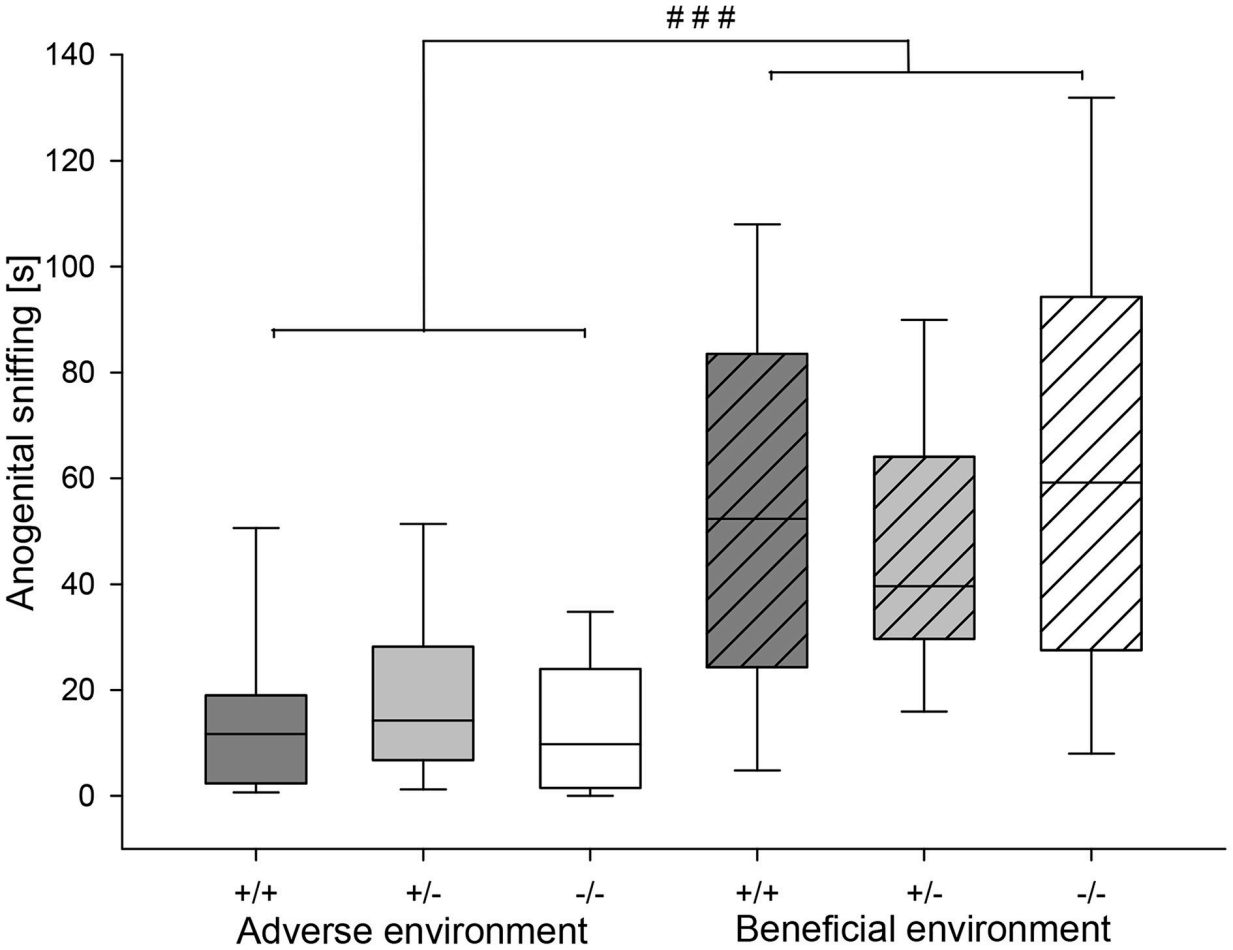
**Duration of anogenital sniffing (in seconds) in the RI test of male heterozygous (+∕−) and homozygous 5-HTT knockout (−∕−) mice and their wildtype counterparts (+∕+) that experienced either an adverse or beneficial environment during adolescence**. Values are presented as box plots with medians, 25–75% quartiles and 10–90% ranges. Effect of social experience: ^###^*p* ≤ 0.001. AE +∕+ n = 13, AE +∕− n = 13, AE −∕− n = 12, BE +∕+ n = 12, BE +∕− n = 13, BE −∕− n = 12.

Agonistic behavior was strongly influenced by social experience: Attack latency was significantly different between different social experiences (*U* = 483.0, *df* = 1, *p* = 0.019). AE mice had a shorter attack latency than BE mice (Figure 5A). Moreover, AE animals showed a significantly higher rate of attack (Figure 5B) and sustained attack (attack: *U* = 434.0, *df* = 1, *p* = 0.004; sustained attack: *U* = 472.5, *df* = 1, *p* = 0.01). Moreover, AE mice showed a trend toward more escalated fighting than BE (*U* = 557.5, *p* = 0.084). On the other hand, chasing was not influenced by social experience (*U* = 599.0, *df* = 1, *p* = 0.231; Table 3). Concerning effects of experience on social interest behavior animals that experienced adverse conditions showed less anogenital sniffing (*U* = 199.5, *df* = 1, *p* < 0.001; Figure 6) and less following behavior than BE animals (*U* = 307.5, *df* = 1, *p* < 0.001). Social experience did not influence nasonasal sniffing (*U* = 643.0, *df* = 1, *p* = 0.528) and the frequency at which the focal animal approached (*U* = 568.5, *df* = 1, *p* = 0.155), was approached (*U* = 581.0, *df* = 1, *p* = 0.196) or was followed (*U* = 611.5, *df* = 1, *p* = 0.298; Table 3).

**Table 3:** **Medians with 25–75 % quartiles of social interest and agonistic behavior during the RI test of male heterozygous (+∕−) and homozygous (−∕−) 5-HTT knockout mice and their wildtype counterparts (+∕+) that experienced either an adverse (AE) or beneficial (BE) environment during adolescence**.

**Resident intruder test**	**AE**			**BE**			**Effect of environment (E)**	**Effect of genotype (G)**
**Parameter**	**+∕+**	**+∕−**	**−∕−**	**+∕+**	**+∕−**	**−∕−**		
**SOCIAL INTEREST BEHAVIOR**								
Anogenital sniffing (s)	12.20 (2.20; 19.55)	13.50 (6.30; 31.70)	9.75 (1.50; 23.98)	52.35 (24.33; 83.55)	39.60 (29.65; 64.05)	59.20 (27.53; 94.3)	***U* = 199.50; *df* = 1; *p* < 0.001**	*X*^2^ = 0.099; *df* = 2: *p* = 0.952
Nasal sniffing (s)	17.00 (9.50; 41.00)	20.00 (13.50; 29.00)	17.50 (9.75; 27.25)	16.00 (13.25; 22.00)	14.00 (12.00; 27.00)	27.50 (20.75; 35.5)	*U* = 643.0; *df* = 1; *p* = 0.528	*X*^2^ = 1.225; *df* = 2; *p* = 0.542
Following (s)	1.00 (0.00; 4.00)	2.00 (0.50; 6.00)	0.00 (0.00; 2.75)	4.00 (1.50; 8.75)	5.00 (2.00; 6.50)	11.50 (7.25; 20.00)	***U* = 307.5; *df* = 1; *p* < 0.001**	*X*^2^ = 0.593; *df* = 2; *p* = 0.744
Being followed (s)	0.00 (0.00; 6.00)	0.00 (0.00; 19.50)	0.00 (0.00; 1.75)	0.00 (0.00; 0.75)	1.00 (0.00; 8.50)	5.00 (1.25; 14.50)	*U* = 611.5; *df* = 1; *p* = 0.298	*X*^2^ = 3.435; *df* = 2; *p* = 0.180
Approaching (#)	23.00 (17.00; 25.50)	21.00 (19.50; 31.50)	17.50 (13.75; 21.50)	19.50 (17.00; 28.25)	20.00 (14.50; 23.50)	16.00 (13.25; 20.75)	*U* = 568.5; *df* = 1; *p* = 0.199	***X*^2^ = 6.949; *df* = 2; *p* = 0.031**
Being approached (#)	4.00 (2.50; 7.50)	5.00 (2.50; 9.00)	5.00 (2.00; 10.00)	1.50 (1.00; 4.00)	4.00 (1.00; 9.50)	4.50 (3.00; 8.00)	*U* = 581.0; *df* = 1; *p* = 0.196	*X*^2^ = 2.546; *df* = 2; *p* = 0.280
**AGONISTIC BEHAVIOR**								
Attack latency (s)	126.30 (82.45; 266.70)	186.60 (109.35; 425.70)	347.75 (314.73; 469.43)	295.35 (207.25; 388.35)	231.70 (139.45; 520.80)	553.55 (371.65; 600.00)	***U* = 483.0; *df* = 1; *p* = 0.019**	***X*^2^ = 14.335; *df* = 2; *p* = 0.001**
Attacking (#)	18.00 (8.50; 32.50)	17:00 (2.50; 28.50)	10.00 (3.00; 22.25)	11.00 (5.25; 22.00)	4.00 (1.00; 19.50)	1.00 (0.00; 6.50)	***U* = 434.5; *df* = 1; *p* = 0.004**	***X*^2^ = 7.354; *df* = 2; *p* = 0.025**
Sustained attack (#)	7.00 (1.50; 10.50)	1.00 (0.00; 6.50)	2.50 (0.00; 7.25)	2.00 (0.00; 9.00)	0.00 (0.00; 7.00)	0.00 (0.00; 0.00)	***U* = 472.5; *df* = 1; *p* = 0.010**	***X*^2^ = 6.632; *df* = 2; *p* = 0.036**
Escalated fighting (#)	2.00 (0.00; 4.50)	0.00 (0.00; 3.00)	0.00 (0.00; 1.00)	0.00 (0.00; 2.00)	0.00 (0.00; 1.50)	0.00 (0.00; 0.00)	*U* = 557.5; *df* = 1; *p* = 0.084	*X*^2^ = 5.257; *df* = 2; *p* = 0.071
Chasing (s)	0.50 (0.00; 4.45)	0.60 (0.00; 3.35)	0.00 (0.00; 2.53)	0.40 (0.00; 2.15)	0.00 (0.00; 3.10)	0.00 (0.00; 0.00)	*U* = 599.0; *df* = 1; *p* = 0.231	*X*^2^ = 5.251; *df* = 2; *p* = 0.072

*Effects of experience (E) and genotype (G) on aggressive and social interest behavior. Statistics: Mann-Whitney U Test for effects of environment and Kruskal-Wallis Test for genotype effects; bold: p ≤ 0.05; AE +∕+ n = 13, AE +∕− n = 13, AE −∕− n = 12, BE +∕+ n = 12, BE +∕− n = 13, BE −∕− n = 12*.

#### Effects of 5-HTT genotype on agonistic behavior

Agonistic behavior was affected by genotype as well. Attack latency time was significantly different between genotypes (*X*^2^ = 14.335, *df* = 2, *p* = 0.001; Figure 5A). Pairwise comparison revealed that 5-HTT −∕− knockout mice had significantly longer latencies than the other two genotypes (5-HTT −∕− vs. 5-HTT +∕+: *p* = 0.002, 5-HTT −∕− vs. 5-HTT +∕−: *p* = 0.004, Bonferroni corrected). Moreover, genotype significantly affected number of attack (*X*^2^ = 7.354, *df* = 2, *p* = 0.025) and sustained attack (*X*^2^ = 6.632, *df* = 2, *p* = 0.036). Pairwise comparison identified significantly fewer attacks and sustained attacks performed by 5-HTT −∕− mice than 5-HTT +∕+ mice (Attack: 5-HTT −∕− vs. 5-HTT +∕+: *p* = 0.007, sustained attack: 5-HTT −∕− vs. 5-HTT +∕+: *p* = 0.012, Bonferroni corrected; Figure 5B). 5-HTT +∕− animals did not differ significantly from either 5-HTT +∕+ or 5-HTT −∕− individuals (Attack: 5-HTT +∕− vs. 5-HTT +∕+: *p* = 0.262, 5-HTT +∕− vs. 5-HTT −∕−: *p* = 0.106; sustained attack: 5-HTT +∕− vs. 5-HTT +∕+: *p* = 0.160, 5-HTT +/- vs. 5-HTT −∕−: *p* = 0.185). Additionally, there was a trend for a difference in escalated fighting between genotypes (*X*^2^ = 5.257, *df* = 2, *p* = 0.071) and chasing behavior (*X*^2^ = 5.251, *df* = 2, *p* = 0.072). Genotype significantly influenced approach behavior (*X*^2^ = 6.949, *df* = 2, *p* = 0.031). However, following Bonferroni correction no significant differences between the genotypes could be detected. The other social interest behaviors were not significantly influenced by genotype (Table 3).

### Effects of social experience and genotype on endocrinological parameters

Ten minutes after the resident intruder test AE animals showed higher levels of plasma corticosterone [*F*_(1, 72)_ = 6.99; *p* = 0.010; Table 4]. Plasma testosterone levels were not significantly affected by social experience (*U* = 718.0; *df* = 1; *p* = 0.680; Table 4). Concerning adrenal tyrosine hydroxylase activity, AE animals showed a trend toward higher activity compared to BE mice [*F*_(1, 71)_ = 3.803; *p* = 0.055; Table 4]. Neither plasma corticosterone levels, nor plasma testosterone levels, nor tyrosine hydroxylase activity were affected by genotype [corticosterone: *F*_(2, 72)_ = 0.520; *p* = 0.597; testosterone: *X*^2^ = 0.319; *df* = 2; *p* = 0.853; TH: *F*_(2, 71)_ = 0.165; *p* = 0.848; Table 4]. No interaction between social experience and genotype was detected for plasma corticosterone levels and tyrosine hydroxylase activity [corticosterone: *F*_(2, 72)_ = 1.415; *p* = 0.250; TH: *F*_(2, 71)_ = 0.121; *p* = 0.886; Table 4].

**Table 4:** **Group means ± SEM of plasma corticosterone concentrations and tyrosine hydroxylase activities and medians with 25–75 % quartiles of plasma testosterone concentrations of male heterozygous (+∕−) and homozygous (−∕−) 5-HTT knockout mice and their wildtype counterparts (+∕+) that experienced either an adverse (AE) or beneficial (BE) environment during adolescence**.

**Endocrinological parameters**	**AE**			**BE**			**Effect of environment (E)**	**Effect of genotype (G)**	**G × E**
	**+∕+**	**+∕−**	**−∕−**	**+∕+**	**+∕−**	**−∕−**			
Plasma corticosterone (ng/ml)	145.72 ± 13.33	121.48 ± 11.18	124.59 ± 10.27	101.25 ± 14.58	116.10 ± 11.45	98.35 ± 7.98	***F*_(1, 72)_ = 6.990; *p* = 0.010**	*F*_(2, 72)_ = 0.520; *p* = 0.597	*F*_(2, 72)_ = 1.415; *p* = 0.250
Tyrosine hydroxylase (nmol/h/adrenal)	1.76 ± 0.25	1.85 ± 0.16	1.91 ± 0.19	1.57 ± 0.14	1.49 ± 0.15	1.61 ± 0.15	*F*_(1, 71)_ = 3.803; p = 0.055	*F*_(2, 71)_ = 0.165; p = 0.848	*F*_(2, 71)_ = 0.121; *p* = 0.886
Plasma testosterone (ng/ml)	0.43 (0.15; 5.42)	0.59 (0.23; 8.62)	0.79 (0.16; 4.95)	0.75 (0.26; 11.47)	0.34 (0.20; 3.23)	1.21 (0.33; 6.88)	*U* = 718.0; *df* = 1; *p* = 0.680)	*X*^2^ = 0.319; *df* = 2; *p* = 0.853	–

*Effects of experience (E) and genotype (G) on aggressive and social interest behavior. Statistics: plasma corticosterone and tyrosine hydroxylase activity: ANOVA; plasma testosterone: Mann-Whitney U Test for effects of environment and Kruskal-Wallis Test for genotype effects; bold: p ≤ 0.05; plasma corticosterone and testosterone AE +∕+ n = 13, AE +∕− n = 14, AE −∕− n = 13, BE +∕+ n = 13, BE +∕− n = 13, BE −∕− n = 12; tyrosine hydroxylase: AE +∕+ n = 13, AE +∕− n = 14, AE −∕− n = 12, BE +∕+ n = 13, BE +∕− n = 13, BE −∕− n = 12. Due to non-parametric testing, no gene by environment interaction can be given for plasma testosterone concentrations*.

## Discussion

In the present study we investigated how different social experiences during adolescence together with varying levels of the 5-HTT gene affect anxiety-like and aggressive behavior in male mice. Remarkably, animals that experienced social adversity exhibited lower levels of anxiety-like behavior compared to individuals that lived in constant beneficial conditions, namely cohabitation with a female partner. Moreover, experiencing adversity during adolescence also led to increased aggressiveness toward a docile intruder. Irrespective of social experience, animals lacking the 5-HTT were more anxious and less aggressive than their heterozygous knockout or wildtype counterparts.

### Social experience during adolescence affects anxiety-like and exploratory behavior

Mice that experienced social adversity during adolescence showed lower levels of anxiety-like behavior as measured by the latency to enter and the time spent in the light compartment during the DL and the time spent in the center of the OF. Moreover, they showed higher levels of exploratory behavior compared to animals that experienced beneficial conditions. This increase could be found in the total distance the animals covered in the EPM and OF and the number of entries to the lit compartment of the DL. It seems surprising that adverse experiences lead to lower levels of anxiety-like behavior than beneficial experiences as these findings contradict other studies that show that adversity itself has anxiogenic effects in rodents (Buwalda et al., 2005). Already a single defeat can increase anxiety-like behavior of rats (Meerlo et al., 1996; Ruis et al., 1999). Moreover, also repeated or chronic adversity has been found to increase anxiety-like behavior in a variety of tests and paradigms in adult as well as adolescent rodents (e.g., Kinsey et al., 2007; Jansen et al., 2010; Jung et al., 2014; Chaby et al., 2015).

One might argue that the adverse experiences in the present study were not sufficient to induce anxiety-like behavior. However, animals that experienced escapable social adversity showed a decreased body weight gain, a clear indicator that the environment was experienced as stressful (Tamashiro et al., 2004, 2007; Rygula et al., 2005; Iñiguez et al., 2014). Moreover, it could already be shown that the encounter with a dominant conspecific in the custom-made cage system that is also used in the present study, leads to significantly increased levels of fecal corticosterone metabolites (Bodden et al., 2015), another indicator for the adversity of the environment. Thus, the results in the present study might rather be explained by the kind of adversity the animals experienced. The main difference to the previously mentioned studies is the “escapability” of the stressor. In the present study the animals could terminate the aversive stimulus by escaping to a refuge cage. It is well known that unescapable or uncontrollable stressors elicit anxiety-like behavior while controllable stressors do not (Korte et al., 1999; Korte and De Boer, 2003; Koolhaas et al., 2011). In accordance with our findings, predictable chronic mild restraint stress decreases anxiety-like behavior in adult (Parihar et al., 2011) and adolescent rats (Suo et al., 2013). Similarly, Kubala and colleagues showed that adolescent rats experiencing escapable tailshocks exhibit higher levels of social exploration compared to rats encountering inescapable tailshocks and control animals (Kubala et al., 2012). Therefore, the nature of the adversity seems to be of major importance in shaping the state anxiety of an animal.

In a previous study using a similar design to ours, it was found that animals that experienced escapable adversity during adulthood showed profoundly reduced levels of anxiety-like behavior (Bodden et al., 2015) However, this was only the case if they had previously experienced mating opportunities (beneficial conditions). If the animals had encountered loser experiences (adverse conditions) their anxiety-like behavior was not reduced after escapable adversity. Counterintuitively, levels were in fact as high as those of animals that experienced only beneficial environments throughout the experiment (Bodden et al., 2015). Thus, animals that experienced beneficial conditions and afterwards had to cope with challenge showed a marked reduction in anxiety-like behavior. Similar to this study, animals of the present study were confronted with escapable social adversity but this time during the phase of adolescence. Previous to this experience, animals lived with their parents and siblings and after weaning in brother groups. In addition to this social enrichment, the animals were also provided with structural enrichment; overall good housing conditions for laboratory mice (Palanza et al., 2001; Bartolomucci et al., 2002; Marashi et al., 2003, 2004). Therefore, prior to experiencing escapable social adversity they did not experience any adversity. Again, escapable social adversity significantly reduced anxiety-like behavior compared to beneficial conditions. It thus seems that rather than a particular experience, the timing and sequence of adverse and beneficial events is of significant importance for shaping anxiety-like behavior.

Whereas social experience during adolescence did influence state anxiety, it did not affect trait anxiety. State anxiety is the anxiety an individual experiences at a certain moment and is augmented by threatening stimuli while trait anxiety is the general tendency of an individual to display anxious behavior (Lister, 1990; Griebel et al., 1993). In the FE, the animal can freely choose if it wants to explore a new environment or stay in the safety of its home cage. The test has been pharmacologically validated (Belzung and Berton, 1997) and is thus thought to be suitable for depicting trait anxiety in mice (Griebel et al., 1993). The present results indicate that trait anxiety is mainly influenced by genetic components (e.g., 5-HTT genotype) and is not significantly altered by experiences of an individual during adolescence. While it has been shown that trait anxiety can be altered by early experience (Kloke et al., 2013), it could be speculated that the phase of adolescence is already too far into the development of an individual to fundamentally change trait anxiety. Interestingly, the effects of social environment were more or less the same for all three genotypes. Currently, we have no good explanation for this missing interaction between experience and genotype.

### 5-HTT genotype affects anxiety-like and exploratory behavior

Furthermore, as expected, we found consistent genotype effects in the EPM, DL, OF, and FE. Homozygous 5-HTT knockout mice showed increased levels of state as well as trait anxiety and a decrease in exploratory behavior compared to their heterozygous and wildtype counterparts. These results are fully in line with previous studies that found differences in state anxiety (e.g., Holmes et al., 2003; Kalueff et al., 2007; Jansen et al., 2010; Heiming et al., 2011; Kloke et al., 2013) and trait anxiety (e.g., Kloke et al., 2013) in mice varying in 5-HTT genotype. It has been suggested that low levels of 5-HTT during development lead to modulations of brain systems involved in emotion and stress regulation which in turn might explain the increased levels of anxiety-like behavior in homozygous 5-HTT knockout mice (Ansorge et al., 2004).

### Social experience during adolescence affects aggressive behavior

Aggressive behaviors—represented by a short attack latency, a high number of attacks and sustained attacks—were augmented after the experience of escapable social adversity. Moreover, animals that experienced escapable social adversity showed lower levels of social interest behavior as measured by anogenital sniffing and following of the docile intruder compared to animals that experienced beneficial conditions.

The increase in aggression of animals that experienced escapable social adversity is in one way surprising, as repeated loser experiences are associated with a downregulation of aggressiveness (Hsu et al., 2006; Kloke et al., 2011). On the other hand, the present findings are in line with several studies showing an increase in aggressive behavior following social adversity (e.g., Veenema et al., 2006; Márquez et al., 2013; Cumming et al., 2014). Alternatively, methodological considerations might explain the obtained results: to induce territoriality, mice from both groups/conditions were housed individually for one week before the Resident Intruder test. This means, the animals experienced two different situations. Animals that previously experienced escapable social adversity no longer underwent this subordination. Animals that experienced a beneficial environment lost their female partner. Therefore, it might not be the experienced environment during adolescence but rather the distinct perception of the individual housing that led to the differences in aggressive behavior. In any case, the social experience during adolescence affected the three genotypes in a comparable manner. Again, no interaction of experience and genotype could be detected.

Concerning endocrinological parameters, higher levels of aggressive behavior during the Resident Intruder test of animals that experienced escapable social adversity was accompanied by increased levels of plasma corticosterone levels and a trend for heightened adrenal tyrosine hydroxylase activity. This indicates an increased activation of the hypothalamic-pituitary axis and the sympatho-adrenomedullary system, respectively. This relationship between hormones and behavior is well established (e.g., Ely and Henry, 1978; Sachser and Lick, 1991; Sachser et al., 1994; Jansen et al., 2011; Pérez-Tejada et al., 2013).

### Effect of 5-HTT genotype on aggressive behavior

Social interest behavior was not majorly influenced by 5-HTT genotype. Only approach behavior was significantly affected in such a way that homozygous 5-HTT knockout mice approached the intruder less often than heterozygous 5-HTT knockout and wildtype mice. This is in line with previous studies (Holmes et al., 2002; Kloke et al., 2011). Contrary, agonistic behavior was strongly influenced by 5-HTT genotype. Homozygous 5-HTT knockout mice had a significantly longer attack latency time than both heterozygous knockout and wildtype mice. Moreover, wildtype mice displayed a higher number of attacks and sustained attacks than homozygous knockout mice. Furthermore a trend toward a difference in escalated fighting and chasing was detected. Of the three genotypes, homozygous knockout mice showed the lowest amount of these behaviors. These findings are in line with the notion that homozygous 5-HTT knockout mice show little agonistic behavior (Holmes et al., 2002; Lewejohann et al., 2010; Jansen et al., 2011; Heiming et al., 2013; but see Kloke et al., 2011). The serotonergic system plays a vital role in the regulation of aggressive behavior. High levels of aggression and violence are associated with low levels of serotonin metabolite levels in the cerebrospinal fluid in humans (Brown and Linnoila, 1990; Linnoila and Virkkunen, 1992; Berman et al., 1997; Coccaro et al., 1997a,b) and animals (Caramaschi et al., 2007; Audero et al., 2013). In the same direction, increased levels of central 5-HT decreases aggression (Gibbons et al., 1978). The serotonin transporter is a key regulator of 5-HT neurotransmission as it takes 5-HT back up from the extracellular space. It was shown that homozygous 5-HTT knockout mice (Mathews et al., 2004) and rats (Homberg et al., 2007) which lack the transporter, have increased levels of 5-HT in the extracellular space. This might account for a decrease in aggressive behavior compared to wildtype and heterozygous knockout mice. One might speculate about the link between aggressive and anxiety-like behavior. In the literature there are some indications that anxiety-induced aggressiveness exists (Prior et al., 2004). In our study however, this seems unlikely since high anxiety in homozygous 5-HTT knockout mice coincides with low aggressiveness, also supporting recent findings (Jansen et al., 2011). Whether there was a causal relationship between anxiety and aggressiveness cannot be concluded on the basis of our data.

### Conclusion

The present study clearly shows that adolescence is a phase where anxiety and aggressiveness can be shaped profoundly by social experiences. Notably, not the experience of throughout beneficial social conditions during adolescence but rather being used and able to cope with challenge results in lower levels of anxiety-like behavior. Taken together, these data suggests that the sequence and timing of beneficial and adverse experiences are crucial for the shaping of the anxious and aggressive behavioral profile.

## Author contributions

Substantial contributions to the conception or design of the work (NM, HR, RS, VK, SK, NS); or the acquisition (NM), analysis (NM), or interpretation of data for the work (NM, HR, RS, VK, SK, KPL, NS); and drafting the work (NM) or revising it critically for important intellectual content (NM, HR, RS, VK, SK, KPL, NS); and final approval of the version to be published (NM, HR, RS, VK, SK, KPL, NS); and agreement to be accountable for all aspects of the work in ensuring that questions related to the accuracy or integrity of any part of the work are appropriately investigated and resolved (NM, HR, RS, VK, SK, KPL, NS).

### Conflict of interest statement

The authors declare that the research was conducted in the absence of any commercial or financial relationships that could be construed as a potential conflict of interest.

## References

[B1] Anholt R. R. H. Mackay T. F. C. (2012). Genetics of aggression. Annu. Rev. Genet. 46, 145–164. 10.1146/annurev-genet-110711-155514 22934647

[B2] Ansorge M. S. Zhou M. Lira A. Hen R. Gingrich J. A. (2004). Early-life blockade of the 5-HT transporter alters emotional behavior in adult mice. Science 306, 879–881. 10.1126/science.1101678 15514160

[B3] Araragi N. Lesch K. P. (2013). Serotonin (5-HT) in the regulation of depression-related emotionality: insight from 5-HT transporter and tryptophan hydroxylase-2 knockout mouse models. Curr. Drug Targets 14, 549–570. 10.2174/1389450111314050005 23547810

[B4] Audero E. Mlinar B. Baccini G. Skachokova Z. K. Corradetti R. Gross C. (2013). Suppression of serotonin neuron firing increases aggression in mice. J. Neurosci. 33, 8678–8688. 10.1523/JNEUROSCI.2067-12.2013 23678112 PMC6618819

[B5] Bartolomucci A. Palanza P. Parmigiani S. (2002). Group housed mice: are they really stressed? Ethol. Ecol. Evol. 14, 341–350. 10.1080/08927014.2002.9522735

[B6] Belzung C. Berton F. (1997). Further pharmacological validation of the BALB/c neophobia in the free exploratory paradigm as an animal model of trait anxiety. Behav. Pharmacol. 8, 541–548. 10.1097/00008877-199711000-00012 9832968

[B7] Bengel D. Murphy D. L. Andrews A. M. Wichems C. H. Feltner D. Heils A.. (1998). Altered brain serotonin homeostasis and locomotor insensitivity to 3, 4-methylenedioxymethamphetamine (“Ecstasy”) in serotonin transporter-deficient mice. Mol. Pharmacol. 53, 649–655. 9547354 10.1124/mol.53.4.649

[B8] Berman M. E. Tracy J. I. Coccaro E. F. (1997). The serotonin hypothesis of aggression revisited. Clin. Psychol. Rev. 17, 651–665. 10.1016/S0272-7358(97)00039-1 9336689

[B9] Bodden C. Richter S. H. Schreiber R. S. Kloke V. Gerß J. Palme R.. (2015). Benefits of adversity?! How life history affects the behavioral profile of mice varying in serotonin transporter genotype. Front. Behav. Neurosci. 9:47. 10.3389/fnbeh.2015.00047 25784864 PMC4347490

[B10] Brown G. L. Linnoila M. I. (1990). CSF serotonin metabolite (5-HIAA) studies in depression, impulsivity, and violence. J. Clin. Psychiatry 51(Suppl.), 31–41; discussion 42–43. 1691169

[B11] Buwalda B. Kole M. H. P. Veenema A. H. Huininga M. de Boer S. F. Korte S. M.. (2005). Long-term effects of social stress on brain and behavior: a focus on hippocampal functioning. Neurosci. Biobehav. Rev. 29, 83–97. 10.1016/j.neubiorev.2004.05.005 15652257

[B12] Calatayud F. Coubard S. Belzung C. (2004). Emotional reactivity in mice may not be inherited but influenced by parents. Physiol. Behav. 80, 465–474. 10.1016/j.physbeh.2003.10.001 14741231

[B13] Caldji C. Tannenbaum B. Sharma S. Francis D. Plotsky P. M. Meaney M. J. (1998). Maternal care during infancy regulates the development of neural systems mediating the expression of fearfulness in the rat. Proc. Natl. Acad. Sci. U.S.A. 95, 5335–5340. 10.1073/pnas.95.9.5335 9560276 PMC20261

[B14] Canli T. Lesch K.-P. (2007). Long story short: the serotonin transporter in emotion regulation and social cognition. Nat. Neurosci. 10, 1103–1109. 10.1038/nn1964 17726476

[B15] Caramaschi D. de Boer S. F. Koolhaas J. M. (2007). Differential role of the 5-HT1A receptor in aggressive and non-aggressive mice: an across-strain comparison. Physiol. Behav. 90, 590–601. 10.1016/j.physbeh.2006.11.010 17229445

[B16] Carola V. Frazzetto G. Pascucci T. Audero E. Puglisi-Allegra S. Cabib S.. (2008). Identifying molecular substrates in a mouse model of the serotonin transporter x environment risk factor for anxiety and depression. Biol. Psychiatry 63, 840–846. 10.1016/j.biopsych.2007.08.013 17949690

[B17] Carroll J. C. Boyce-Rustay J. M. Millstein R. Yang R. Wiedholz L. M. Murphy D. L.. (2007). Effects of mild early life stress on abnormal emotion-related behaviors in 5-HTT knockout mice. Behav. Genet. 37, 214–222. 10.1007/s10519-006-9129-9 17177116

[B18] Carver C. S. Johnson S. L. McCullough M. E. Forster D. E. Joormann J. (2014). Adulthood personality correlates of childhood adversity. Front. Psychol. 5:1357. 10.3389/fpsyg.2014.01357 25484874 PMC4240049

[B19] Caspi A. Sugden K. Moffitt T. E. Taylor A. Craig I. W. Harrington H.. (2003). Influence of life stress on depression: moderation by a polymorphism in the 5-HTT gene. Science 301, 386–389. 10.1126/science.1083968 12869766

[B20] Chaby L. E. Cavigelli S. A. Hirrlinger A. M. Caruso M. J. Braithwaite V. A. (2015). Chronic unpredictable stress during adolescence causes long-term anxiety. Behav. Brain Res. 278, 492–495. 10.1016/j.bbr.2014.09.003 25448433

[B21] Coccaro E. F. Berman M. E. Kavoussi R. J. (1997a). Assessment of life history of aggression: development and psychometric characteristics. Psychiatry. Res. 73, 147–157. 10.1016/S0165-1781(97)00119-4 9481806

[B22] Coccaro E. F. Kavoussi R. J. Trestman R. L. Gabriel S. M. Cooper T. B. Siever L. J. (1997b). Serotonin function in human subjects: intercorrelations among central 5-HT indices and aggressiveness. Psychiatry Res. 73, 1–14. 10.1016/S0165-1781(97)00108-X 9463834

[B23] Coccaro E. F. Lee R. Kavoussi R. J. (2010). Inverse relationship between numbers of 5-HT transporter binding sites and life history of aggression and intermittent explosive disorder. J. Psychiatry Res. 44, 137–142. 10.1016/j.jpsychires.2009.07.004 19767013

[B24] Cumming M. J. Thompson M. A. McCormick C. M. (2014). Adolescent social instability stress increases aggression in a food competition task in adult male Long-Evans rats. Dev. Psychobiol. 56, 1575–1588. 10.1002/dev.21252 25176514

[B25] Ely D. L. Henry J. P. (1978). Neuroendocrine response patterns in dominant and subordinate mice. Horm. Behav. 10, 156–169. 10.1016/0018-506X(78)90005-3 29002

[B26] Ferrari P. F. Palanza P. Parmigiani S. de Almeida R. M. M. Miczek K. A. (2005). Serotonin and aggressive behavior in rodents and nonhuman primates: predispositions and plasticity. Eur. J. Pharmacol. 526, 259–273. 10.1016/j.ejphar.2005.10.002 16289029

[B27] Francis D. (1999). Nongenomic Transmission Across Generations of Maternal Behavior and Stress Responses in the Rat. Science 286, 1155–1158. 10.1126/science.286.5442.1155 10550053

[B28] Frankle W. G. Lombardo I. New A. S. Goodman M. Talbot P. S. Huang Y. (2005). Brain serotonin transporter distribution in subjects with impulsive aggressivity: a positron emission study with [11C]McN 5652. Am. J. Psychiatry 162, 915–923. 10.1176/appi.ajp.162.5.915 15863793

[B29] Gibbons J. L. Barr G. A. Bridger W. H. Leibowitz S. F. (1978). Effects of para-chlorophenylalanine and 5-hydroxytryptophan on mouse killing behavior in killer rats. Pharmacol. Biochem. Behav. 9, 91–98. 10.1016/0091-3057(78)90017-5 151866

[B30] Griebel G. Belzung C. Misslin R. Vogel E. (1993). The free-exploratory paradigm: an effective method for measuring neophobic behaviour in mice and testing potential neophobia-reducing drugs. Behav. Pharmacol. 4, 637–644. 10.1097/00008877-199312000-00009 11224232

[B31] Gross C. Hen R. (2004). The developmental origins of anxiety. Nat. Rev. Neurosci. 5, 545–552. 10.1038/nrn1429 15208696

[B32] Haller J. Harold G. Sandi C. Neumann I. D. (2014). Effects of adverse early-life events on aggression and anti-social behaviours in animals and humans. J. Neuroendocrinol. 26, 724–738. 10.1111/jne.12182 25059307

[B33] Haller J. Kruk M. R. (2006). Normal and abnormal aggression: human disorders and novel laboratory models. Neurosci. Biobehav. Rev. 30, 292–303. 10.1016/j.neubiorev.2005.01.005 16483889

[B34] Heiming R. S. Bodden C. Jansen F. Lewejohann L. Kaiser S. Lesch K.-P.. (2011). Living in a dangerous world decreases maternal care: a study in serotonin transporter knockout mice. Horm. Behav. 60, 397–407. 10.1016/j.yhbeh.2011.07.006 21787775

[B35] Heiming R. S. Mönning A. Jansen F. Kloke V. Lesch K.-P. Sachser N. (2013). To attack, or not to attack? The role of serotonin transporter genotype in the display of maternal aggression. Behav. Brain Res. 242, 135–141. 10.1016/j.bbr.2012.12.045 23291155

[B36] Heiming R. S. Sachser N. (2010). Consequences of serotonin transporter genotype and early adversity on behavioral profile - pathology or adaptation? Front. Neurosci. 4:187. 10.3389/fnins.2010.00187 21151780 PMC2999984

[B37] Hennessy M. B. Kaiser S. Tiedtke T. Sachser N. (2015). Stability and change: stress responses and the shaping of behavioral phenotypes over the life span. Front. Zool. 12:S18. 10.1186/1742-9994-12-S1-S18 26816517 PMC4722350

[B38] Holmes A. Murphy D. L. Crawley J. N. (2002). Reduced aggression in mice lacking the serotonin transporter. Psychopharmacology (Berl). 161, 160–167. 10.1007/s00213-002-1024-3 11981596

[B39] Holmes A. Yang R. J. Lesch K.-P. Crawley J. N. Murphy D. L. (2003). Mice lacking the serotonin transporter exhibit 5-HT(1A) receptor-mediated abnormalities in tests for anxiety-like behavior. Neuropsychopharmacology 28, 2077–2088. 10.1038/sj.npp.1300266 12968128

[B40] Homberg J. R. Olivier J. D. A. Smits B. M. G. Mul J. D. Mudde J. Verheul M.. (2007). Characterization of the serotonin transporter knockout rat: a selective change in the functioning of the serotonergic system. Neuroscience 146, 1662–1676. 10.1016/j.neuroscience.2007.03.030 17467186

[B41] Hovatta I. Barlow C. (2008). Molecular genetics of anxiety in mice and men. Ann. Med. 40, 92–109. 10.1080/07853890701747096 18293140

[B42] Hovens J. G. F. M. Wiersma J. E. Giltay E. J. van Oppen P. Spinhoven P. Penninx B. W. J. H.. (2010). Childhood life events and childhood trauma in adult patients with depressive, anxiety and comorbid disorders vs. controls. Acta Psychiatry Scand. 122, 66–74. 10.1111/j.1600-0447.2009.01491.x 19878136

[B43] Hsu Y. Earley R. L. Wolf L. L. (2006). Modulation of aggressive behaviour by fighting experience: mechanisms and contest outcomes. Biol. Rev. Camb. Philos. Soc. 81, 33–74. 10.1017/S146479310500686X 16460581

[B44] Iñiguez S. D. Riggs L. M. Nieto S. J. Dayrit G. Zamora N. N. Shawhan K. L.. (2014). Social defeat stress induces a depression-like phenotype in adolescent male c57BL/6 mice. Stress 17, 247–255. 10.3109/10253890.2014.910650 24689732 PMC5534169

[B45] Ishikawa J. Nishimura R. Ishikawa A. (2015). Early-life stress induces anxiety-like behaviors and activity imbalances in the medial prefrontal cortex and amygdala in adult rats. Eur. J. Neurosci. 41, 442–453. 10.1111/ejn.12825 25581710

[B46] Jansen F. Heiming R. S. Kloke V. Kaiser S. Palme R. Lesch K.-P.. (2011). Away game or home match: the influence of venue and serotonin transporter genotype on the display of offensive aggression. Behav. Brain Res. 219, 291–301. 10.1016/j.bbr.2011.01.029 21262270

[B47] Jansen F. Heiming R. S. Lewejohann L. Touma C. Palme R. Schmitt A.. (2010). Modulation of behavioural profile and stress response by 5-HTT genotype and social experience in adulthood. Behav. Brain Res. 207, 21–29. 10.1016/j.bbr.2009.09.033 19782704

[B48] Jung Y. H. Hong S. I. Ma S. X. Hwang J. Y. Kim J. S. Lee J. H.. (2014). Strain differences in the chronic mild stress animal model of depression and anxiety in mice. Biomol. Ther. (Seoul). 22, 453–459. 10.4062/biomolther.2014.058 25414777 PMC4201223

[B49] Kaiser S. Sachser N. (1998). The social environment during pregnancy and lactation affects the female offsprings' endocrine status and behaviour in guinea pigs. Physiol. Behav. 63, 361–366. 10.1016/S0031-9384(97)00435-6 9469727

[B50] Kalueff A. V. Fox M. A. Gallagher P. S. Murphy D. L. (2007). Hypolocomotion, anxiety and serotonin syndrome-like behavior contribute to the complex phenotype of serotonin transporter knockout mice. Genes. Brain. Behav. 6, 389–400. 10.1111/j.1601-183X.2006.00270.x 16939636

[B51] Karg K. Burmeister M. Shedden K. Sen S. (2011). The serotonin transporter promoter variant (5-HTTLPR), stress, and depression meta-analysis revisited: evidence of genetic moderation. Arch. Gen. Psychiatry 68, 444–454. 10.1001/archgenpsychiatry.2010.189 21199959 PMC3740203

[B52] Kaufman J. Yang B. Z. Douglas-Palumberi H. Houshyar S. Lipschitz D. Krystal J. H.. (2004). Social supports and serotonin transporter gene moderate depression in maltreated children. Proc. Natl. Acad. Sci. U.S.A. 101, 17316–17321. 10.1073/pnas.0404376101 15563601 PMC534414

[B53] Kendler K. S. (1995). The structure of the genetic and environmental risk factors for six major psychiatric disorders in women. Arch. Gen. Psychiatry 52, 374. 10.1001/archpsyc.1995.03950170048007 7726718

[B54] Kinsey S. G. Bailey M. T. Sheridan J. F. Padgett D. A. Avitsur R. (2007). Repeated social defeat causes increased anxiety-like behavior and alters splenocyte function in C57BL/6 and CD-1 mice. Brain. Behav. Immun. 21, 458–466. 10.1016/j.bbi.2006.11.001 17178210 PMC1941837

[B55] Kloke V. Heiming R. S. Bölting S. Kaiser S. Lewejohann L. Lesch K.-P.. (2013). Unexpected effects of early-life adversity and social enrichment on the anxiety profile of mice varying in serotonin transporter genotype. Behav. Brain Res. 247, 248–258. 10.1016/j.bbr.2013.03.039 23567893

[B56] Kloke V. Jansen F. Heiming R. S. Palme R. Lesch K.-P. Sachser N. (2011). The winner and loser effect, serotonin transporter genotype, and the display of offensive aggression. Physiol. Behav. 103, 565–574. 10.1016/j.physbeh.2011.04.021 21549735

[B57] Koolhaas J. M. Bartolomucci A. Buwalda B. de Boer S. F. Flügge G. Korte S. M.. (2011). Stress revisited: a critical evaluation of the stress concept. Neurosci. Biobehav. Rev. 35, 1291–1301. 10.1016/j.neubiorev.2011.02.003 21316391

[B58] Korte S. M. De Boer S. F. (2003). A robust animal model of state anxiety: fear-potentiated behaviour in the elevated plus-maze. Eur. J. Pharmacol. 463, 163–175. 10.1016/S0014-2999(03)01279-2 12600708

[B59] Korte S. M. De Boer S. F. Bohus B. (1999). Fear-potentiation in the elevated plus-maze test depends on stressor controllability and fear conditioning. Stress 3, 27–40. 10.3109/10253899909001110 19016191

[B60] Kubala K. H. Christianson J. P. Kaufman R. D. Watkins L. R. Maier S. F. (2012). Short- and long-term consequences of stressor controllability in adolescent rats. Behav. Brain Res. 234, 278–284. 10.1016/j.bbr.2012.06.027 22771417 PMC3684236

[B61] Lesch K. P. Bengel D. Heils A. Sabol S. Z. Greenberg B. D. Petri S.. (1996). Association of anxiety-related traits with a polymorphism in the serotonin transporter gene regulatory region. Science 274, 1527–1531. 10.1126/science.274.5292.1527 8929413

[B62] Lesch K. P. Merschdorf U. (2000). Impulsivity, aggression, and serotonin: a molecular psychobiological perspective. Behav. Sci. Law 18, 581–604. 10.1002/1099-0798(200010)18:5<581::AID-BSL411>3.0.CO;2-L 11113963

[B63] Lewejohann L. Kloke V. Heiming R. S. Jansen F. Kaiser S. Schmitt A.. (2010). Social status and day-to-day behaviour of male serotonin transporter knockout mice. Behav. Brain Res. 211, 220–228. 10.1016/j.bbr.2010.03.035 20347882

[B64] Linnoila V. M. Virkkunen M. (1992). Aggression, suicidality, and serotonin. J. Clin. Psychiatry 53 (Suppl,) 46–51. 1385390

[B65] Lister R. G. (1990). Ethologically-based animal models of anxiety disorders. Pharmacol. Ther. 46, 321–340. 10.1016/0163-7258(90)90021-S 2188266

[B66] Marashi V. Barnekow A. Ossendorf E. Sachser N. (2003). Effects of different forms of environmental enrichment on behavioral, endocrinological, and immunological parameters in male mice. Horm. Behav. 43, 281–292. 10.1016/S0018-506X(03)00002-3 12694638

[B67] Marashi V. Barnekow A. Sachser N. (2004). Effects of environmental enrichment on males of a docile inbred strain of mice. Physiol. Behav. 82, 765–776. 10.1016/j.physbeh.2004.05.009 15451640

[B68] Márquez C. Poirier G. L. Cordero M. I. Larsen M. H. Groner A. Marquis J.. (2013). Peripuberty stress leads to abnormal aggression, altered amygdala and orbitofrontal reactivity and increased prefrontal MAOA gene expression. Transl. Psychiatry 3, e216. 10.1038/tp.2012.144 23321813 PMC3566724

[B69] Mathews T. A. Fedele D. E. Coppelli F. M. Avila A. M. Murphy D. L. Andrews A. M. (2004). Gene dose-dependent alterations in extraneuronal serotonin but not dopamine in mice with reduced serotonin transporter expression. J. Neurosci. Methods 140, 169–181. 10.1016/j.jneumeth.2004.05.017 15589347

[B70] Meerlo P. Overkamp G. J. F. Daan S. van den Hoofdakker R. H Koolhaas J. M. (1996). Changes in behaviour and body weight following a single or double social defeat in rats. Stress 1, 21–32. 10.3109/10253899609001093 9807059

[B71] Nagatsu T. Levitt M. Udenfriend S. (1964). A rapid and simple radioassay for tyrosine hydroxylase activity. Anal. Biochem. 9, 122–126. 10.1016/0003-2697(64)90092-2 14246117

[B72] Olivier B. (2015). Serotonin: a never-ending story. Eur. J. Pharmacol. 753, 2–18. 10.1016/j.ejphar.2014.10.031 25446560

[B73] Palanza P. Gioiosa L. Parmigiani S. (2001). Social stress in mice: gender differences and effects of estrous cycle and social dominance. Physiol. Behav. 73, 411–420. 10.1016/S0031-9384(01)00494-2 11438369

[B74] Parihar V. K. Hattiangady B. Kuruba R. Shuai B. Shetty A. K. (2011). Predictable chronic mild stress improves mood, hippocampal neurogenesis and memory. Mol. Psychiatry 16, 171–183. 10.1038/mp.2009.130 20010892 PMC2891880

[B75] Pérez-Tejada J. Arregi A. Gómez-Lázaro E. Vegas O. Azpiroz A. Garmendia L. (2013). Coping with chronic social stress in mice: hypothalamic-pituitary-adrenal/ sympathetic-adrenal-medullary axis activity, behavioral changes and effects of antalarmin treatment: implications for the study of stress-related psychopathologies. Neuroendocrinology 98, 73–88. 10.1159/000353620 23796983

[B76] Prior H. Schwegler H. Marashi V. Sachser N. (2004). Exploration, emotionality, and hippocampal mossy fibers in nonaggressive AB/Gat and congenic highly aggressive mice. Hippocampus 14, 135–140. 10.1002/hipo.10166 15058491

[B77] Provençal N. Booij L. Tremblay R. E. (2015). The developmental origins of chronic physical aggression: biological pathways triggered by early life adversity. J. Exp. Biol. 218, 123–133. 10.1242/jeb.111401 25568459

[B78] Reif A. Rösler M. Freitag C. M. Schneider M. Eujen A. Kissling C.. (2007). Nature and nurture predispose to violent behavior: serotonergic genes and adverse childhood environment. Neuropsychopharmacology 32, 2375–2383. 10.1038/sj.npp.1301359 17342170

[B79] Ruis M. A. te Brake J. H. Buwalda B. De Boer S. Meerlo P. Korte S.. (1999). Housing familiar male wildtype rats together reduces the long-term adverse behavioural and physiological effects of social defeat. Psychoneuroendocrinology 24, 285–300. 10.1016/S0306-4530(98)00050-X 10101734

[B80] Rygula R. Abumaria N. Flügge G. Fuchs E. Rüther E. Havemann-Reinecke U. (2005). Anhedonia and motivational deficits in rats: impact of chronic social stress. Behav. Brain Res. 162, 127–134. 10.1016/j.bbr.2005.03.009 15922073

[B81] Sachser N. Hennessy M. B. Kaiser S. (2011). Adaptive modulation of behavioural profiles by social stress during early phases of life and adolescence. Neurosci. Biobehav. Rev. 35, 1518–1533. 10.1016/j.neubiorev.2010.09.002 20854842

[B82] Sachser N. Kaiser S. Hennessy M. B. (2013). Behavioural profiles are shaped by social experience: when, how and why. Philos. Trans. R. Soc. Lond. B. Biol. Sci. 368, 20120344. 10.1098/rstb.2012.0344 23569292 PMC3638447

[B83] Sachser N. Lick C. (1991). Social experience, behavior, and stress in guinea pigs. Physiol. Behav. 50, 83–90. 10.1016/0031-9384(91)90502-F 1946736

[B84] Sachser N. Lick C. Stanzel K. (1994). The environment, hormones, and aggressive behaviour: a 5-year-study in guinea pigs. Psychoneuroendocrinology 19, 697–707. 10.1016/0306-4530(94)90051-5 7938365

[B85] Spear L. P. (2000). The adolescent brain and age-related behavioral manifestations. Neurosci. Biobehav. Rev. 24, 417–463. 10.1016/S0149-7634(00)00014-2 10817843

[B86] Sugden K. Arseneault L. Harrington H. Moffitt T. E. Williams B. Caspi A. (2010). Serotonin transporter gene moderates the development of emotional problems among children following bullying victimization. J. Am. Acad. Child Adolesc. Psychiatry 49, 830–840. 10.1016/j.jaac.2010.01.024 20643316 PMC2908591

[B87] Suo L. Zhao L. Si J. Liu J. Zhu W. Chai B.. (2013). Predictable chronic mild stress in adolescence increases resilience in adulthood. Neuropsychopharmacology 38, 1387–1400. 10.1038/npp.2013.67 23478858 PMC3682155

[B88] Tamashiro K. L. K. Hegeman M. A. Nguyen M. M. N. Melhorn S. J. Ma L. Y. Woods S. C.. (2007). Dynamic body weight and body composition changes in response to subordination stress. Physiol. Behav. 91, 440–448. 10.1016/j.physbeh.2007.04.004 17512562 PMC1986729

[B89] Tamashiro K. L. K. Nguyen M. M. N. Fujikawa T. Xu T. Yun Ma L. Woods S. C.. (2004). Metabolic and endocrine consequences of social stress in a visible burrow system. Physiol. Behav. 80, 683–693. 10.1016/j.physbeh.2003.12.002 14984803

[B90] Tsoory M. Cohen H. Richter-Levin G. (2007). Juvenile stress induces a predisposition to either anxiety or depressive-like symptoms following stress in adulthood. Eur. Neuropsychopharmacol. 17, 245–256. 10.1016/j.euroneuro.2006.06.007 16889944

[B91] Veenema A. H. Blume A. Niederle D. Buwalda B. Neumann I. D. (2006). Effects of early life stress on adult male aggression and hypothalamic vasopressin and serotonin. Eur. J. Neurosci. 24, 1711–1720. 10.1111/j.1460-9568.2006.05045.x 17004935

[B92] Wei L. David A. Duman R. S. Anisman H. Kaffman A. (2010). Early life stress increases anxiety-like behavior in Balb c mice despite a compensatory increase in levels of postnatal maternal care. Horm. Behav. 57, 396–404. 10.1016/j.yhbeh.2010.01.007 20096699 PMC2849915

[B93] Witte P. U. Matthaei H. (1980). Mikrochemische Methoden für Neurobiologische Untersuchungen. Berlin: Springer.

[B94] Wommack J. C. Taravosh-Lahn K. David J. T. Delville Y. (2003). Repeated exposure to social stress alters the development of agonistic behavior in male golden hamsters. Horm. Behav. 43, 229–236. 10.1016/S0018-506X(02)00029-6 12614654

